# Integral Membrane Protein 2A Is a Negative Regulator of Canonical and Non-Canonical Hedgehog Signalling

**DOI:** 10.3390/cells10082003

**Published:** 2021-08-06

**Authors:** Cintli C. Morales-Alcala, Ioanna Ch. Georgiou, Alex J. Timmis, Natalia A. Riobo-Del Galdo

**Affiliations:** 1 Leeds Institute of Medical Research, University of Leeds, Leeds LS2 9JT, UK; bs15ccma@leeds.ac.uk (C.C.M.-A.); ioanna.georgiou@liverpool.ac.uk (I.C.G.); 2 School of Molecular and Cellular Biology, University of Leeds, Leeds LS2 9JT, UK; bs15at@leeds.ac.uk

**Keywords:** hedgehog, patched1, ITM2A, GLI, autophagy, skeletal muscle

## Abstract

The Hedgehog (Hh) receptor PTCH1 and the integral membrane protein 2A (ITM2A) inhibit autophagy by reducing autolysosome formation. In this study, we demonstrate that ITM2A physically interacts with PTCH1; however, the two proteins inhibit autophagic flux independently, since silencing of ITM2A did not prevent the accumulation of LC3BII and p62 in PTCH1-overexpressing cells, suggesting that they provide alternative modes to limit autophagy. Knockdown of ITM2A potentiated PTCH1-induced autophagic flux blockade and increased PTCH1 expression, while ITM2A overexpression reduced PTCH1 protein levels, indicating that it is a negative regulator of PTCH1 non-canonical signalling. Our study also revealed that endogenous ITM2A is necessary for timely induction of myogenic differentiation markers in C2C12 cells since partial knockdown delays the timing of differentiation. We also found that basal autophagic flux decreases during myogenic differentiation at the same time that ITM2A expression increases. Given that canonical Hh signalling prevents myogenic differentiation, we investigated the effect of ITM2A on canonical Hh signalling using GLI-luciferase assays. Our findings demonstrate that ITM2A is a strong negative regulator of GLI transcriptional activity and of GLI1 stability. In summary, ITM2A negatively regulates canonical and non-canonical Hh signalling.

## 1. Introduction

The Hedgehog (Hh) signalling pathway has essential functions in embryonic development, tissue homeostasis and cancer [1]. PTCH1 is the most important Hh receptor and a well-established tumour suppressor [2]. The canonical signalling pathway is initiated by binding of one of the Hh ligands (Shh, Ihh, or Dhh) to the 12-transmembrane receptor Patched1 (PTCH1). In the absence of Hh ligands, PTCH1 represses the activation of the G protein-coupled receptor Smoothened (SMO), resulting in processing of the GLI2 and GLI3 transcription factors into repressors. Binding of a Hh protein to PTCH1 inhibits its intrinsic activity, believed to be the transport of cholesterol across the plasma membrane to generate a localized gradient, leading to derepression of SMO and its accumulation at the primary cilium. Ciliary SMO induces activation of GLI2 and GLI3 and prevents their processing, initiating a GLI-dependent transcriptional response that results in upregulation of the short-lived strong activator GLI1 and of the Hh protein-sequestering membrane proteins PTCH1, PTCH2 and HHIP, among other cell type-specific targets.

More recent studies have revealed that PTCH1 has additional functions independently of SMO/GLI, known as “Type I non-canonical Hh signalling” to differentiate it from SMO-dependent, GLI-independent processes [2,3]. The C-terminal domain (CTD) of PTCH1 is dispensable for canonical Hh signalling [4,5], although one study suggests that the CTD is necessary for PTCH1 localization to the primary cilium [6]. The first identified role of the CTD was a pro-apoptotic function, mediated by interaction with the DRAL/TUCAN/Caspase9 complex and independent of SMO [7,8,9,10]. With the goal of identifying additional proteins that interact with the CTD of PTCH1, we previously performed a yeast-2-hybrid study, in which we identified the autophagy-related protein ATG101, a subunit of the ULK complex that mediates initiation of autophagy [11]. Our study established that the cytosolic CTD of PTCH1 inhibits autophagic flux by impairment of autophagosome-lysosome fusion or acidification and that its interaction with ATG101 was important for that function. Paradoxically, ATG101 regulates autophagy initiation and expansion of phagophore membranes to form the autophagosome and no role in the terminal step of autophagy has been discovered. This led us to hypothesize that PTCH1 might block autophagy completion with the aid of additional interacting proteins. In this study, we focus on the potential regulatory role of integral membrane protein 2A (ITM2A), which was identified in a large proportion of our positive clones in the yeast-2-hybrid screen. Remarkably, out of 63 sequenced and validated positive hits, ITM2A was recovered in 21 of them (33%). A novel function of ITM2A in blocking autophagic flux by an inhibitory interaction with v-ATPase was reported a few years ago [12], suggesting a potential functional interaction with PTCH1. However, the role of ITM2A in autophagy remains controversial since it was proposed to induce autophagy in breast cancer cells [13].

ITM2A is a type II single-pass membrane protein with intracellular N-terminal domain and an extracellular BRICHOS domain [14]. Two alternatively spliced forms of ITM2A were described: the long isoform of 263 aa (30 kDa) and a shorter isoform of 219 aa lacking the transmembrane domain (25 kDa). Although the function of ITM2A is not completely elucidated, it has been linked to differentiation of skeletal muscle, cartilage tissue and T cell development [15,16]. Early reports indicate that ITM2A levels increase during skeletal muscle differentiation and the formation of myotubes in vivo and in C2C12 cells, a model of in vitro myogenesis [15,17,18]. Overexpression of ITM2A can accelerate myotube formation; however, it is not essential for muscle development, as indicated by in vitro knockdown studies and in conditional knockout mice [17,18]. Canonical Hh signalling prevents myogenic differentiation by keeping C2C12 and primary satellite muscle cells in the proliferative state [19]. In particular, Gli1 and Gli2 were shown to repress MyoD-dependent transcription, preventing terminal myotube differentiation [20]. This suggests that ITM2A function could be inversely correlated to canonical Hh signalling activity.

Given the involvement of ITM2A in biological processes that are regulated themselves by different types of Hh signalling and the potential physical interaction with PTCH1, we decided to investigate their crosstalk.

## 2. Materials and Methods

### 2.1. Reagents

The antibodies used in this study (name, catalog number, vendor and dilution used in Western blotting) is described in Supplementary Table S1. Bafilomycin A1 was from Tocris (cat. 1334). Acrylamide: bisacrylamide (19:1) solution (cat. 1610154) and TEMED (cat. 1610801) were from BioRad (Watford, UK). All other reagents were research grade and purchased from Fisher Scientific (Loughborough, UK) or Merck Life Science (Gillingham, UK).

### 2.2. Cell Lines

HEK293 (cat. CRL-1573), HeLa cells (cat. CCL-2), and C2C12 cells (ATCC cat. CRL-1772) were purchased from the American Type Culture Collection (Manassas, Virginia). NIH3T3 cells (93061524) were obtained from the European Collection of Authenticated Cell Cultures (Salisbury, UK). *Ptch1*^−/−^ mouse embryonic fibroblasts were a gift from Dr. Matthew Scott (Stanford University).

### 2.3. Cell Culture

HEK293 and HeLa cells were maintained in DMEM (Merck, cat. D6546) containing 10% fetal bovine serum (Gibco cat. 10270106) and penicillin/streptomycin in a humidified 5% CO_2_ incubator. In preparation for transfection, cells were seeded in antibiotic-free media into culture dishes of different areas and transfected with Lipofectamine 2000 when they reached 80–90% confluency, following the manufacturer’s instructions. To generate shRNA stable cells, cells were transduced with lentiviral particles, encoding scrambled or ITM2A targeting shRNA sequences (Table 1), produced in HEK293T cells as previously described [21].

C2C12 cells were cultured in DMEM containing 10% fetal bovine serum and penicillin/streptomycin in a humidified 5% CO_2_ incubator. In order to keep the cells in the undifferentiated state, they were split when reaching 70% confluency. Differentiation was induced by changing the medium of 100% confluent cultures by DMEM containing 2% horse serum (Sigma cat. H1270) and penicillin/streptomycin. The day the differentiation medium was added was called day 0, and the cells were kept for 7 days with media refresh every other day. The generation of stable shRNA ITM2A and control C2C12 cells was performed by transduction of undifferentiated cells with lentiviruses encoding hairpin RNAs (Table 1), followed by selection with puromycin.

### 2.4. Quantitative PCR

Total RNA was isolated from each of the three cell lines with RNeasy Mini Kit (QIAGEN, Manchester, UK; cat 74104) and quantified by A260nm/A280nm in a Nanodrop. Synthesis of cDNA was performed using 1 μg RNA with iScript cDNA Synthesis Kit (BioRad) using hexarandom primers following the manufacturer’s instructions. Real-time quantitative PCR was performed using 1–5 μL of cDNA and target-specific primers (Table 2) with the SsoFast EvaGreen Supermix (BioRad) with a CFX Connect Real-Time PCR Detection System (BioRad) thermocycler. Amplification was quantified and expressed as fold using the ΔΔCt method.

### 2.5. Co-Immunoprecipitation

HEK293 were plated in a 10 cm culture dish and transiently transfected using Lipofectamine^TM^ 2000 (Thermo Fisher, Waltham, MA, USA; cat. 11668030) using 20 μL transfection reagent and 8 μg total DNA. For co-immunoprecipitation (co-IP) assays, two different plasmids were used in the same reaction (4 µg of each plasmid). Twenty-four hours after transfection, cells were washed with 2 mL of ice-cold 1× PBS and scraped in 700 µL of co-IP lysis buffer (50 mM Tris HCl pH 7.5, 150 mM NaCl, 1% NP-40, 0.05% Sodium deoxycholate, 1 mM EDTA, 2.5 Mm MgCl_2_) supplemented immediately before use with 1× Proteoloc protease inhibitor cocktail (Gentaur, London, UK; cat. 44204), 0.2 mM PMSF and 1 mM DTT. Lysates were incubated at 4 °C for 30 min with rotation and then centrifuged at 13,000 RPM for 15 min at 4 °C. The supernatant was separated and 200 µL set apart as whole cell lysate (WCL) and mixed with 40 µL of 6× Laemmli buffer. The remaining supernatant was incubated with 4 μg primary antibody (Table 3) at 4 °C with rotation for 1.5 h, followed by the addition of 30 µL Dynabeads Protein G (Thermo Fisher; cat. 10003D) for an additional 1 h incubation at 4 °C with rotation. A magnetic rack was used to collect and wash the beads three times with 1 mL of co-IP lysis buffer. Immunoprecipitates were extracted from the bead by the addition of 18 µL of 2× Laemmli buffer. Both beads and WCL were incubated on heat block at 45 °C for 25 min and stored at −80 °C for Western blotting.

### 2.6. Gli-Luciferase Assay

NIH3T3 cells and *Ptch1*^−/−^ mouse embryonic fibroblasts (MEFs) were used for this assay. Cells were grown in a 10 cm culture dish to an approximate confluence of 90%, trypsinised, and seeded in 24-well plates at a 1:5 dilution in growth media. Cells were transfected 24 h after plating using different transfection reagents for each cell line: FuGENE^®^HD (Promega, Southampton, UK; cat. E2311) for NIH3T3 and TransIT-X2^®^ (Mirus Bio, Madison, WI, USA; cat MIR6003) for MEFs.

Assays were always conducted in triplicate. For each well, a fixed amount of p8xGBS-Luc (135 ng), pRL-SV40 or pRL-TK (15 ng/well) and testing plasmid DNA (375 ng, containing a single plasmid or a combination of plasmids) and 1.5 µL of the transfection reagent were used, following the manufacturer’s instructions. After 24 h, transfected cells were incubated with serum starvation media (DMEM, 0.5% FBS, 1% GlutaMAX) for an additional 48 h. Cells were washed with 0.5 mL of PBS and lysed with 100 µL passive lysis buffer with vigorous shaking at room temperature for 15 min. Dual-luciferase reporter assay system (Promega; cat. E1910) was used to measure the activity of *Firefly*-luciferase and *Renilla*-luciferase using a Promega GloMax 20/20 luminometer (cat. E5311). Values of the individual measurements were generated as relative luciferase units (RLUs) and as *Firefly*-luciferase/ *Renilla*-luciferase ratio.

### 2.7. Mass Spectrometry

To prepare the samples for proteomic analysis, 3 × 10^5^ HEK293 cells were seeded in 10 cm culture dishes and transiently transfected 24 h later with HA-ITM2A or empty pcDNA3.1 plasmid, using 8 µg of DNA and 20 µL of Lipofectamine^TM^ 2000. Twenty-four hours after transfection, cells were washed with 2 mL of ice-cold 1x PBS and processed for IP as described above. Samples were submitted in solution form to the mass spectrometry facility of the University of Leeds for the identification of immunoprecipitation-interacting protein partners and for the detection of protein post-translational modifications. Protein digestion of samples was carried out with trypsin. Data was processed by the facility using the software Peaks (Bioinformatics Solutions, Waterloo, ON, Canada).

### 2.8. STRING Protein Association Network

An interactome map was constructed using STRING for the ITM2A-specific identified proteins by mass spectrometry analysis. The interactome was constructed with the STRING database using default settings; the confidence score was set to medium (0.4) with network edges showing the confidence of an interaction.

### 2.9. Statistical Analysis

GraphPad Prism 9 was used for the statistical analysis and for the generation of graphs. Unless otherwise specified, three biological replicates were performed. Error bars are shown as standard error of the mean (SEM). Two-tailed paired Student’s *t*-test was used to analyse the significant difference between two groups. One-way ANOVA analysis was selected to analyse at least three different groups when the samples showed normal distribution and equal variance.

## 3. Results

### 3.1. ITM2A Physically Interacts with the Hedgehog Receptor PTCH1

**Figure 1:**
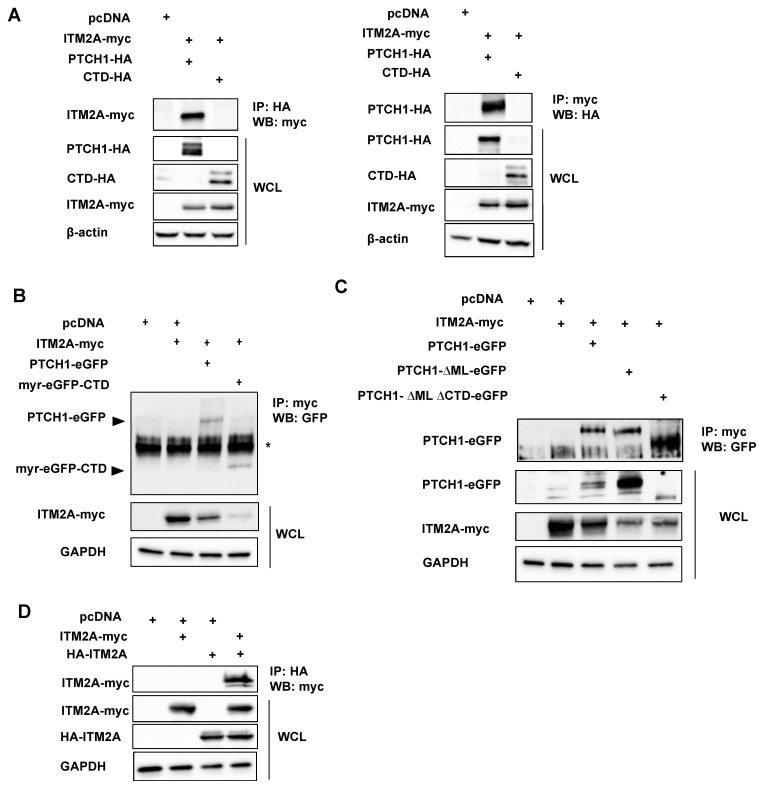
ITM2A and PTCH1 interact in mammalian cells. (**A**) HEK293 cells were transfected with PTCH1-HA or CTD-HA with or without ITM2A-myc and a fraction of the whole cell lysate (WCL) was immunoprecipitated with an anti-HA antibody and blotted with an anti-myc antibody (left), or immunoprecipitated with anti-myc and developed with anti-HA (right). (**B**) HEK293 cells were transfected with ITM2A-myc and eGFP-tagged PTCH1 or a construct encoding myristoylated-CTD, followed by immunoprecipitation using anti-myc for detection of GFP-PTCH1 fragments (indicated by arrowheads). Asterisk (*) signals a crossreactive band. (**C**) Co-expression of ITM2A-myc with deletion mutants of PTCH1 tagged with eGFP, followed by immunoprecipitation with anti-myc and blotting with anti-GFP. (**D**) HEK293 transfected with myc-tagged ITM2A and HA-tagged ITM2A or empty vector were subjected to immunoprecipitation with anti-HA followed by blotting with anti-myc. All experiments were repeated 3 times with similar results.

Following the interaction studies in yeast cells, we tested if human PTCH1 interacts with ITM2A in mammalian cells by co-immunoprecipitation. First, we determined that full-length PTCH1-HA was capable of interacting with myc-tagged ITM2A when co-expressed in HEK293 cells. Reciprocal pulldowns using anti-myc or anti-HA antibodies confirmed the interaction in mammalian cells (Figure 1A). Unexpectedly, the CTD alone (aa 1176–1447) in soluble form was unable to interact strongly with ITM2A (Figure 1A). Since the yeast-2-hybrid experiment that initially indicated an interaction of ITM2A with PTCH1 used only the CTD as bait, we considered the possibility that for interaction of the proteins in mammalian cells, co-localisation at the plasma membrane was required. Therefore, we expressed a membrane-associated CTD (through fusion with a myristoylated GFP (CAAX-eGFP-CTD) [10] and compared the interaction to ITM2A with full-length PTCH1-eGFP or empty myr-eGFP as a negative control. Immunoprecipiation of myc-ITM2A resulted in detection of full length PTCH1 and the CTD fragment, indicating that membrane-associated CTD was sufficient for their interaction (Figure 1B).

To determine which domains of PTCH1 are necessary for PTCH1-ITM2A physical interaction, we generated PTCH1 mutants with deletions of the two largest intracellular domains, the middle loop (ML) and the CTD [5]. Removal of the ML alone (PTCH1ΔML) or both ML and CTD (PTCH1ΔMLΔCTD) did not prevent ITM2A interaction, indicating that, in addition to the CTD, the transmembrane and/or extracellular loops of PTCH1 independently interact with presumably different regions of the transmembrane protein ITM2A (Figure 1C).

PTCH1 has been recently shown to form stable dimers even in the absence of Hh ligands [22]. We reasoned that ITM2A binding to the CTD and additional domains could cluster together two molecules of ITM2A. Thus, we tested if ITM2A is competent to form self-interactions, as such could be permissive for the formation of higher order magnitude complexes with PTCH1 dimers. As shown in Figure 1D, ITM2A forms strong interactions with itself, supporting a stoichiometric model of 2 PTCH1: 2 ITM2A molecules in the complex.

### 3.2. ITM2A Does Not Mediate PTCH1 Autophagic Flux Inhibition

**Figure 2:**
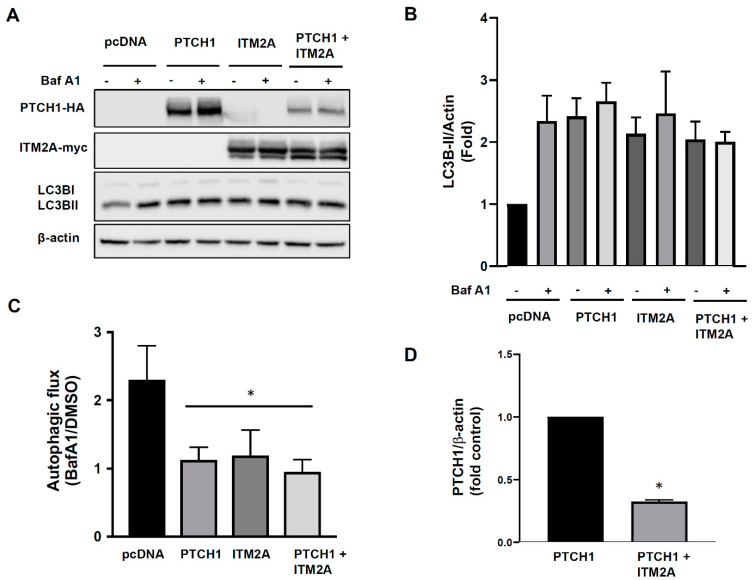
Overexpression of ITM2A does not enhance inhibition of autophagic flux by PTCH1. (**A**) Expression levels of LC3BI and LC3BII in HEK293 cells transfected with pcDNA3.1, PTCH1-HA, myc-ITM2A or both and cultured 24 h in complete growth medium with or without the addition of 100 nM Bafilomycin A1 (BafA1) during the last 4 h. Representative experiment of *n* = 3. (**B**) Densitometric quantification of LC3BII/β-actin ratio in the indicated conditions (*n* = 3). Ratio of pcDNA-transfected cells in the absence of BafA1 was set to 1. (**C**) Autophagic flux index (LC3BII with BafA1 over LC3BII without BafA1) in the indicated conditions and normalized to pcDNA3.1 (*n* = 3; * *p* < 0.05). (**D**) Densitometric quantification of protein levels of PTCH1 24 h post-transfection, normalised to β-actin, in the absence and presence of ITM2A (*n* = 3; * *p* < 0.01).

Since ITM2A was also reported to reduce autophagic flux via interaction with the vacuolar vATPase [12] and we confirmed that ITM2A is a bona fide PTCH1 CTD interactor, we hypothesised that ITM2A could be a mediator of non-canonical PTCH1 signalling in autophagy. Transient transfection of PTCH1 or ITM2A alone increased the levels of LC3BII, the lipidated form of LC3B, a marker of autophagosome formation. However, addition of Bafilomycin A1 during the last 4 h did not increase LC3BII in cells expressing PTCH1 or ITM2A, unlike in cells transfected with empty vector, suggesting that it is the result of LC3BII accumulation as a consequence of an autophagic flux blockade at the last step of the autophagosome-lysosome fusion (Figure 2A,B). Co-expression of PTCH1 and ITM2A had a very similar effect (Figure 2A,B), although we observed a reproducible reduction of PTCH1 expression level when co-expressed with ITM2A (Figure 2A,D), which was not associated with changes in PTCH1 ubiquitylation (Figure S2). Densitometry-based analysis of the magnitude of the Bafilomycin A1 effect revealed that autophagic flux was reduced by more than 80% in all groups (Figure 2C).

**Figure 3:**
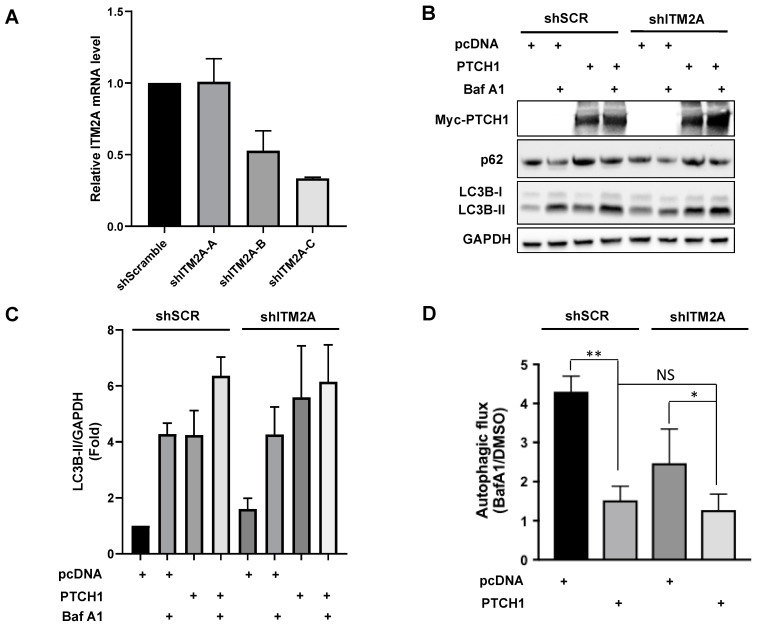
ITM2A silencing has little effect on the autophagic function of PTCH1 in HEK293 cells. (**A)** Relative ITM2A mRNA level normalised to GAPDH in HEK293 cell clones stably transfected with different shRNAs targeting ITM2A. Mean +/- SEM of 3 independent qPCR measurements. (**B**) Expression levels LC3BII in HEK293 shScrambled (shSCR) and HEK293 shITM2A-A (shITM2A) cells transfected with pcDNA or myc-PTCH1, and cultured 24 h in complete growth medium with or without the addition of 100 nM Bafilomycin (BafA1) during the last 4 h. Representative experiment of *n* = 3. (**C**) Densitometric quantification of LC3BII Western blot signals in A normalized to GAPDH in pcDNA (*n* = 3). (**D**) Calculated values of Autophagic flux (ratio of LC3BII with BafA1 over LC3BII with vehicle) from C (*n* = 3; ** *p* = 0.007; * *p* < 0.1; NS, non-significant).

Since the lack of an additive effect of PTCH1 and ITM2A could be the result of maximal inhibition of autophagic flux by overexpression of either of the two proteins, we decided to study the effect of silencing endogenous ITM2A using shRNA. HEK293 cells were transfected with shITM2A and selected with puromycin to generate multiclonal cells; however, ITM2A expression was restored after several passages (data not shown). This might be because as a heterogeneous population, cells expressing higher levels of ITM2A had a growth advantage and their proportion increased during passaging. Given this problem, we isolated shITM2A single cell-derived clones using the limiting dilution method. Three different shRNA sequences targeting ITM2A and a control sequence (shScrambled) were used and clones with the highest knockdown efficiency that was maintained for several passages were used for the following experiments. Clone shITM2A-C maintained a minimum of 65% knockdown (Figure 3A).

Transient expression of PTCH1 in shITM2A-A HEK293 cells showed a small but significant increase in LC3BII and p62 (an autophagy scaffolding protein that is degraded along with the cargo) accumulation by PTCH1 compared to control shScramble cells (Figure 3B,C) and a larger inhibition of autophagic flux (Figure 3D). The enhancing effect of ITM2A depletion on PTCH1-dependent autophagy regulation was also observed in HeLa cells (Supplementary Figure S1A,B). Interestingly, silencing of ITM2A resulted in a small increase of PTCH1 expression levels (Figure 2A and Supplementary Figure S1A). Altogether, these findings suggest that ITM2A is not necessary for autophagy regulation by PTCH1, and that it might actually reduce non-canonical PTCH1 signalling by modulating its turnover.

### 3.3. Increase in ITM2A Levels Accompanies Reduction of Autophagic Flux during Skeletal Muscle Differentiation

**Figure 4:**
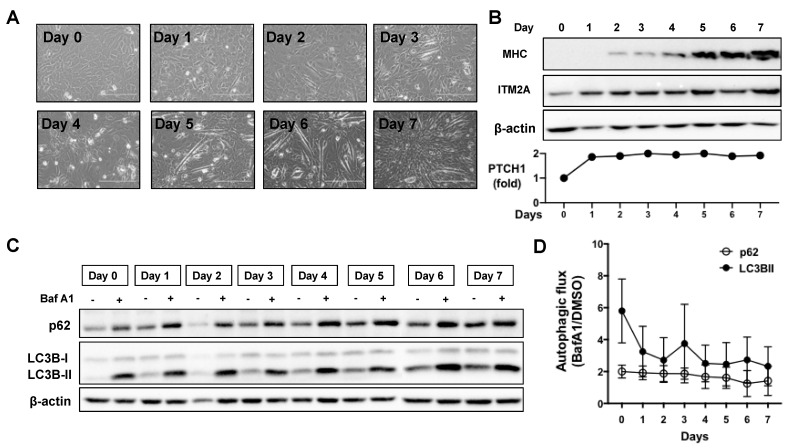
Autophagic flux decreases during myotube differentiation of C2C12 cells. (**A**) Morphological changes of C2C12 from confluent myoblasts (day 0) to multinucleated myotubes (day 7). (**B**) Expression levels of myosin heavy chain (MHC), ITM2A, and PTCH1 (qPCR) in C2C12 cells during 7 days of differentiation. (**C**) Expression levels of p62 and LC3BII in C2C12 cells during 7 days of differentiation day with or without the addition of 100 nM Bafilomycin (BafA1) during the last 4 h. Differentiation media was added to cells at day 0 and changed to fresh differentiation media every other day. Representative experiment of *n* = 3. (**D**) Calculated values of autophagic flux using densitometry of LC3BII and p62 levels normalised to β-actin. Autophagic flux was calculated as the ratio of each marker in the presence of BafA1 over its level with vehicle (DMSO) for each day of differentiation. Graph shows mean +/− SEM, *n* = 3.

Given the involvement of Hh signalling and ITM2A in myotube differentiation of the C2C12 myoblastic cell line, and that ITM2A has been reported to be a marker of skeletal muscle differentiation, we investigated their potential interplay during in vitro differentiation of C2C12 myoblasts into myotubes (Figure 4A). As C2C12 cells differentiate at high density, they upregulate myosin expression from day 3 onwards (Figure 4B). As previously reported [15], ITM2A levels increase significantly during differentiation (Figure 4B). To investigate the regulation of autophagy by endogenous ITM2A and endogenous PTCH1, we first investigated if there were any changes in basal autophagic flux during the 7 days of differentiation of C2C12 cells. The level of p62 and LC3BII increased during differentiation, becoming gradually more insensitive to Bafilomycin A1 treatment (Figure 4C,D). This observation suggests that autophagic flux is reduced in differentiated myotubes compared to proliferating myoblasts.

**Figure 5:**
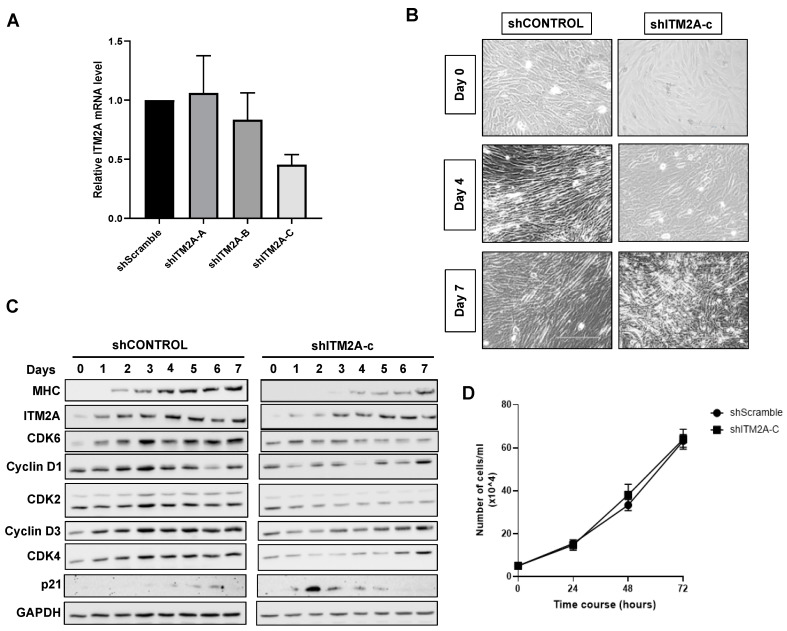
ITM2A knockdown delays differentiation of C2C12 cells. (**A**) Relative expression level of ITM2A in the shRNA clones in undifferentiated C2C12 cells. Graph shows mean +/- SEM normalised to scrambled control, *n* = 3. (**B**) Morphological changes of a control C2C12 clone (shControl) compared to 3 independent shITM2A clones from confluent myoblasts (day 0) to multinucleated myotubes (day 7). (**C**) Expression levels of cell cycle markers during differentiation of control and shITM2A-clone C cells. (**D**) Effect of ITM2A knockdown on undifferentiated C2C12 cells proliferation over 72 h. Graph shows mean +/− SEM, *n* = 3.

We next generated stable C2C12 myoblastic lines with reduced ITM2A expression using three different shRNA sequences. Puromycin-resistant shITM2A cells were analysed for knockdown of ITM2A. Cells stably expressing the shITM2A-B and -C sequences showed 21 and 55% reduction of ITM2A by qPCR, respectively, while shITM2A-A was ineffective (Figure 5B). We continued our study with the shITM2A-C cells, which showed the best knockdown efficiency. C2C12 shITM2A-C cells presented a morphological delay in myotube differentiation (Figure 5A), which was confirmed by delayed myosin upregulation by Western blot, compared to parental and control shScramble C2C12 cells (Figure 5A). Analysis of key cell cycle regulators in shITM2A-C C2C12 cells showed an early increase of p21 at day 3 and a lack of or delayed upregulation of CDK2, CDK4, CDK6, cyclin D1 and cyclin D3 compared to control shCONTROL cells (Figure 5C).

### 3.4. ITM2A Is as a Negative Regulator of Canonical Hh Signalling

**Figure 6:**
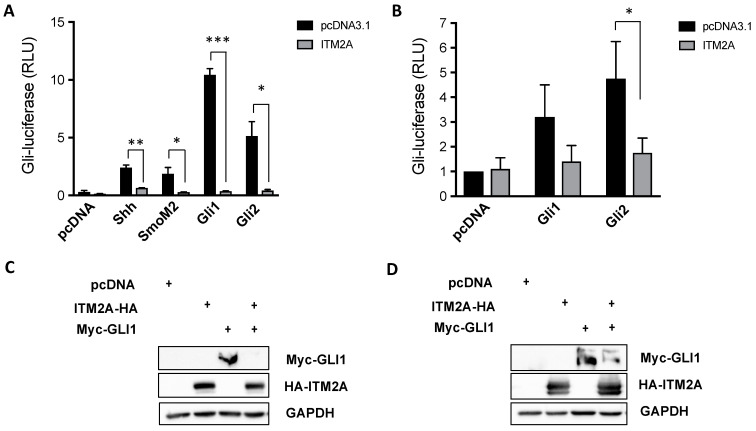
ITM2A is a negative regulator of canonical Hh signalling. (**A**) Relative Gli-luciferase activity (normalized to pRL-TK) in NIH 3T3 cells transfected with the indicated construct combination. Graph shows mean +/- SEM of a representative experiment performed in triplicate (total n=3 independent experiments). * *p* < 0.05; ** *p* < 0.01; *** *p* < 0.0001. (**B**) Same as in A in *Ptch1^−/−^* MEFs (*n* = 3). * *p* < 0.05. (**C**) Effect of ITM2A on total proteins levels of myc-Gli1 in NIH3T3 cells. (**D**) Effect of ITM2A on total proteins levels of myc-Gli1 in HEK293 cells.

Given that we observed a regulatory effect of ITM2A on PTCH1 expression level and non-canonical signalling in autophagy in epithelial cell lines, we next sought to investigate if ITM2A also affects canonical Hh signalling using a well-established GLI-luciferase activity assay in the Hh-responsive NIH 3T3 cells. Remarkably, the overexpression of ITM2A inhibited Gli-luciferase activation by Shh, an oncogenic SMO mutant (SMO-M2), Gli1 and Gli2 (Figure 6A). The same effect was observed in *Ptch1^−/−^* mouse embryonic fibroblasts (MEFs), which display high constitutive canonical Hh signalling activity due to loss of endogenous Ptch1, when overexpressing mouse Gli1 or Gli2 (Figure 6B). Furthermore, ITM2A strongly reduced myc-GLI1 levels when expressed in NIH3T3 cells (Figure 6C), or in HEK293 cells (Figure 6D), a non-ciliated cell type in which GLI-dependent transcription can be stimulated in response to GLI1 overexpression. These findings suggest that ITM2A acts as a negative regulator of Gli stability and transcriptional activity, independently of its interaction with PTCH1.

### 3.5. Identification of ITM2A-Interacting Proteins by Mass Spectrometry

In order to better understand the role of ITM2A in Hh signalling and skeletal muscle differentiation, we overexpressed a tagged ITM2A protein in HEK293 cells and performed immunoprecipitation followed by mass spectrometry to identify potential interacting proteins. Cells expressing empty vector were used as a negative control to subtract non-specific contaminating proteins. ITM2A immunoprecipitates contained a number of proteins listed in Table 4.

**Figure 7:**
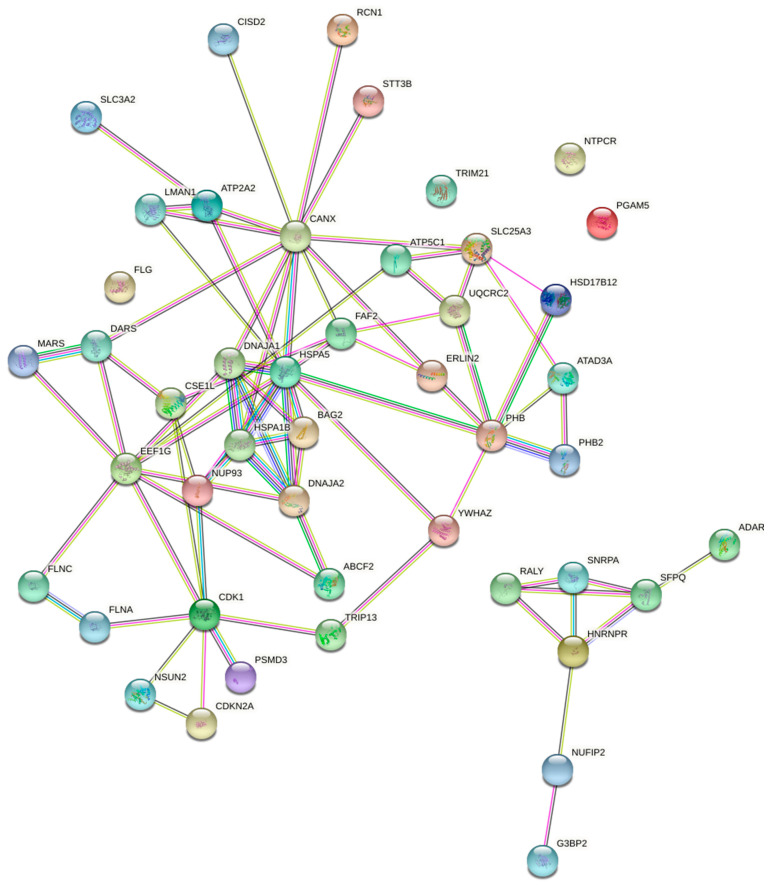
Unsupervised STRING clustering of proteins identified in ITM2A immunoprecipitates from HEK 293 cells. The number of connecting lines increases with experimental evidence of physical or functional interaction. Known interactions = light blue and pink lines; predicted interactions = green, red, and dark blue; text mining = yellow; co-expression = black; protein homology = purple.

STRING analysis reveals a highly significant clustering (*p* = 1 × 10^−16^) of over 90% of the proteins identified (Figure 7), with the existence of sub-clusters related to ER protein quality control, mitochondria membrane protein translocation, nuclear import, and stress granules. ITM2A interaction with NUP93 and CSE1L could be involved in negative regulation of Gli-dependent transcription by the impairment of GLI1 and GLI2 nuclear import. Some other proteins identified play important roles in the recognition of defective mitochondria for clearance by mitophagy (prohibitin and prohibitin-2, ADACT3, HSPA1B). This snapshot of the ITM2A interactome suggests that it may function at ER-mitochondria contacts to regulate mitophagy, which could contribute to mitochondrial network remodeling during myogenic differentiation.

## 4. Discussion

In this study, we present evidence that the single-pass membrane protein ITM2A is a negative regulator of canonical and non-canonical Hh signalling. On the one hand, ITM2A physically interacts with PTCH1 and reduces its stability and biological function as an endogenous inhibitor of autophagy. On the other hand, ITM2A reduces GLI1 stability and inhibits GLI-transcriptional activity at a step downstream of PTCH1, impairing canonical Hh signalling. Our findings refute our original hypothesis that ITM2A could be a mediator of PTCH1-induced autophagic flux reduction, suggesting instead that ITM2A’s effect on autophagy are independent of PTCH1. The identification of ITM2A-interacting proteins by mass spectrometry provides the basis for testable hypotheses of the mechanism by which ITM2A acts as a negative regulator of Hh signalling and how it regulates myogenic differentiation, which will be explored in the future.

In the first part of our study, we confirm that ITM2A interacts with the CTD of PTCH1, and at least with another domain, since a PTCH1 mutant with deletions of the CTD and cytosolic middle loop, was able to co-immunoprecipitate ITM2A. This suggests that ITM2A likely interacts with PTCH1 through its intracellular 53 aa domain and the extracellular BRICHOS domain.

ITM2A and PTCH1 exert the same autophagy blocking phenotype characterised by an accumulation of autophagosomes [11,12]. However, our findings demonstrate that their interaction is not necessary for the autophagic flux blockade induced by PTCH1 in HEK293 or HeLa cells. In agreement with the lack of cooperative effect, the expression of ITM2A causes a direct or indirect degradation of PTCH1, while silencing of endogenous ITM2A results in a concomitant increase in PTCH1 levels. Even when the change in the expression of PTCH1 in the ITM2A knockdown cells was not statistically significant, the slight increase in PTCH1 expression was enough to enhance its inhibitory effect on autophagy. It is possible that the effects on the knockdown are not as marked as the effects of the overexpression because the endogenous levels of ITM2A in HEK293 and HeLa are low, and also because the efficiency of the knockdown was not 100%. This is supported by the observation that, when comparing the effects in the two cells lines, the results are statistically significant in HeLa cells, which presented a higher knockdown efficiency. In summary, our study suggests that ITM2A is not a necessary mediator of the autophagic flux blockade regulated by PTCH1, but instead reduces its activity, while still maintaining its capacity to block autophagy independently of their interaction.

While the effect of ITM2A over PTCH1 protein levels suggested that it could stimulate canonical Hh signalling, GLI-luciferase experiments show a clear inhibitory effect when the pathway is activated by upregulation of ligand (Shh), by an oncogenic SMO mutant, or by the overexpression of the main GLI family transcriptional activators, GLI1 and GLI2. This indicates that ITM2A exerts an inhibitory effect at the level of the GLI transcription factors. While the inhibitory mechanism is unknown, we speculate that interaction of ITM2A with NUP93 (a nucleoporin) and CSE1L (exportin) might regulate GLI2 ciliary trafficking and/or nuclear accumulation. NUP93 has been shown to localise at the ciliary base and regulate the permeability barrier [23]. CSE1L mediates the re-export of importin-α from the nucleus after importin substrates, which include GLI2, are released in the nucleoplasm [24,25].

The negative regulation of ITM2A on Hh signalling could play an important role during myogenic differentiation. It is well known that canonical Shh signalling maintains myoblasts and satellite cells in the proliferative state, when ITM2A levels are lower. Induction of myogenic terminal differentiation is accompanied by upregulation of ITM2A and a simultaneous decrease in autophagic flux. While our interactome analysis in HEK293 cannot be directly extrapolated to other cell types, it showed a subset of proteins that participate in the recognition of defective mitochondria for clearance by mitophagy (prohibitin and prohibitin-2, ADACT3, HSPA1B). This selective type of autophagy has been shown to play an important role during mitochondrial network remodelling in myogenic differentiation and could explain the regulatory role of ITM2A in C2C12 cells.

The process of myogenic differentiation requires the induction of muscle-specific genes, such as myosin, and irreversible cell cycle withdrawal [26,27]. Previous studies showed that the upregulation of cyclin D3, as opposed to cyclin D1 and cyclin D2, plays a key role in cell cycle withdrawal during myogenic differentiation [28,29]. Our results show that knockdown of ITM2A expression in C2C12 cells results in a delay in differentiation together with reduced levels of cyclin D3 expression. Therefore, it is possible that ITM2A regulates a process needed for upregulation of cyclin D3 during differentiation of C2C12 cells. Furthermore, ITM2A could regulate cell cycle progression through interaction with CDKN2A (also known as p16^INK4^), as suggested by our mass spectrometry analysis. Given the positive role of ITM2A in C2C12 differentiation, it is possible that it can stimulate the function of this CDK4/6 inhibitor to accelerate cell cycle exit. Future studies will investigate if ITM2A is involved in stabilization of the main complexes involved in the maintenance of the cell cycle withdrawal required for the differentiation of myoblasts into myotubes. The potential physical interaction with CDKN2 suggested by the proteomics analysis is an attractive starting point to further investigate this process.

In conclusion, the results presented here support a negative role of ITM2A on Hh signalling and confirm its requirement during terminal differentiation of skeletal muscle.

## Figures and Tables

**Table 1:** shRNA sequences used to silence ITM2A in human and mouse cell lines.

Name	Sequence (5′–3′)
ITM2A-A (human)	GATCCCTGCAGCAATTATTCATGATTCAAGGATCATGAATAATTGCTGCAGTTTTT
ITM2A-B (human)	GATCCGGAAATTCGTGATGTTATTTCAAGGAACTAACATCACGAATTTCCTTTTT
ITM2A-C (human)	GATCCCAAGATCTGTCAAGAGTAATTCAAGAGATTACTCTTGACAGATCTTGTTTTT
Itm2a-A (mouse)	GATCCCTAGGCCTCTCATTCATCTTTCAAGAGAAGATGAATGAGAGGCCTAGTTTTT
Itm2a-B (mouse)	GATCCGGATCCTGTCAATTCCATTTTCAAGAGAAATGGAATTGACAGGATCCTTTTT
Itm2a-C (mouse)	GATCCCAAGCGTGCCATTGACAAATTCAAGAGATTTGTCAATGGCACGCTTGTTTTT
Scrambled	Santa Cruz Biotechnology Scrambled shRNA (sc-108060)

NIH3T3 cells and *Ptch1^−/−^* MEFs were maintained in DMEM supplemented with 10% FBS, penicillin-streptomycin and split before they reached 70% confluency.

**Table 2:** qPCR primer sequences.

Name	Species	Sequence (5′–3′)
ITM2A	Human	Forward: ACTGCTATCTGATGCCCCTCAAT Reverse: GGTCTTCTCGAACCACATAAGTTTG
Itm2a	Mouse	Forward: CGCACTGTCCGAGCTCAAAT Reverse: CATCTCCCAGATGAGCCATC
Ptc1	Mouse	Forward: ATGGTCCTGGCTCTGATGAC Reverse: TAGCCCTGTGGTTCTTGTCC
GADPH	Human	Forward: TCCCATCACCATCTTCCA Reverse: CATCACGCCACAGTTTCC
Gapdh	Mouse	Forward: AGTATGATGACATCAAGAAGG Reverse: ATGGTATTCAAGAGAGTAGGG

**Table 3:** Antibodies used for immunoprecipitation.

Antibody	Company	Catalogue
GFP-tag (mouse monoclonal)	Proteintech	66002-1-g
HA-tag (mouse monoclonal)	Proteintech	66006-2-Ig
MYC-tag (rabbit polyclonal)	Proteintech	16286-1-AP

**Table 4:** ITM2A-interacting proteins identified by mass spectroscopy.

Protein Name	Function
ABCF2	ATP-binding cassette transporter
ACTG1	Actin cytoskeleton
ADAR	Adenosine deaminase, RNA editing
ALB	Albumin
ATAD3A	Mitochondrial network organization, steroid hormone
ATP2A2	ER-isolation membrane contacts, autophagosome formation
ATP5C1	Mitochondria -ATP production
ATXN2L	Stress granule and P-body formation
BAG2	Co-chaperone for HSP70/HSC70
C1orf57	Nucleotide phosphatase
CANX	ER protein folding quality control
CDK1	Cell cycle progression
CDKN2A	Negative regulator of proliferation
CISD2	Regulator of autophagy
CSE1L	Nuclear export receptor
DARS	Aspartate tRNA-ligase
DNAJA1	Co-chaperone of HSC70, mitochondria protein transport
DNAJA2	Co-chaperone of HSC70
EEF1G	Elongation factor, protein synthesis
EIF4A1	Eukaryotic initiation factor
ERLIN2	ERAD, cholesterol homeostasis
FAF2	ERAD
FLG	Keratin intermediate filament aggregation
FLNA	Branching of actin filaments
FLNC	Muscle-specific filamin
G3BP2	Stress granule formation
GANAB	Neutral alpha-glucosidase
GP96	ER-chaperone, ERAD
HNRNPR	Processing of mRNA precursor in the nucleus
HSD17B12	ER-localised fatty acid elongation
HSPA1B	HSP70 chaperone
HSPA5	ER chaperone
LMAN1	ER to Golgi transport
MARS	Methionine-tRNA ligase
NPM1	Ribosome biogenesis, ribosome nuclear export
NSUN2	RNA-C5 methyl transferase, tRNA stability, mRNA decay
NUFIP2	Stress granules, transcription control
NUP93	Nuclear pore complex
PAI-1	Serine protease inhibitor
PGAM5	Mitochondrial dynamics
PHB	Mitochondrial chaperone, mitophagy receptor
PHB-2	Mitochondrial chaperone, mitophagy receptor
PSMD3	Component of the 26S proteasome
RALY	Transcriptional cofactor for cholesterogenesis
RCN	ER-post-ER calcium regulatory protein
SFPQ	Pre-mRNA splicing factor, myoblast marker
SLC25A3	Cytosol-mitochondria phosphate transport
SLC3A2	Amino acid transport in plasma membrane and lysosomes
SNRPA	Pre-mRNA splicing factor
STT3B	N-glycosylation of misfolded protein for ERAD
TRIM21	E3 ligase, promotes autophagy, autophagy receptor
TRIP13	Meiosis, chromosomal recombination
UQCRC2	Mitochondrial electron transport chain
YWHAZ	Autophagy regulator

ERAD; endoplasmic reticulum-associated degradation.

## Data Availability

All data related to this study is presented in this manuscript; however, any reasonable request can be directed to N.A.R.-D.G.

## Supplementary Materials

The following are available online at https://www.mdpi.com/article/10.3390/cells10082003/s1, Figure S1: Effect of ITM2A silencing on PTCH function in autophagy in HeLa cells, Figure S2: Effect of ITM2A on PTCH1 ubiquitylation, Table S1: Antibodies used in this study.
