# Supplementary material for: Integral Membrane Protein 2A Is a Negative Regulator of Canonical and Non-Canonical Hedgehog Signalling
Source: Cells. 2021 Aug 6;10(8):2003. doi: 10.3390/cells10082003 (PMC8394137; doi:10.3390/cells10082003)
Supplement: Supplementary file 1 [file cells-10-02003-s001.zip › cells-1307750-supplementary.pdf]

Supplementary Materials

Table S1.

Primary antibody	Species	Dilution	Company	Catalogue number
β-Actin (Monoclonal)	Mouse	1:10,000 in 5% milk	Sigma	A5316
CDK2 (78B2) (Monoclonal)	Rabbit	1:1000 in 5% BSA	CST	2546
CDK4 (DCS156) (Monoclonal)	Mouse	1:1000 in 5% milk	CST	2906
CDK6 (DCS83) (Monoclonal)	Mouse	1:2000 in in 5% milk	CST	3136
Cyclin D1 (DCS6) (Monoclonal)	Mouse	1:1000 in 5% milk	CST	2926
Cyclin D3 (DCS22) (Monoclonal)	Mouse	1:2000 in 5% milk	CST	2936
p21 Cip1/Waf1 (12D1)(Monoclonal)	Rabbit	1:1000 in 5% BSA	CST	2947
DYKDDDDK Tag (D6W5B)	Rabbit	1:1000 in 5% BSA	CST	14793
DYKDDDDK tag (Monoclonal)	Mouse	1:2000 in 5% milk	Proteintech	66008-3-Ig
GFP (4B10) (Monoclonal)	Mouse	1:1000 in 5% milk	CST	2955
GFP tag (Monoclonal)	Mouse	1:4000 in 5% milk	Proteintech	66002-1-g
HA-Tag (C29F4) (Monoclonal)	Rabbit	1:1000 in 5% BSA	CST	3724
Ha tag (Polyclonal)	Rabbit	1:5000 in 5% milk	Proteintech	51064-2-AP
Ha tag (Monoclonal)	Mouse	1:5000 in 5% milk	Proteintech	66006-2-Ig
HRP-conjugated GAPDH (Monoclonal)	Mouse	1:4000 in 5% milk	Proteintech	HRP-60004
ITM2A	Rabbit	1:600 in 5% milk	Proteintech	18306-1-AP
Myc-Tag (9B11) (Monoclonal)	Mouse	1:1000 in 5% milk	CST	2276
MYC-tag (Polyclonal)	Rabbit	1:5000 in 5% milk	Proteintech	16286-1-AP
Myosin Heavy Chain (Monoclonal)	Mouse	0.2μg/ml in 5% milk	DSHB	MF20
LC3B (Polyclonal)	Rabbit	1:1,000 in 5% BSA	CST	2775
SQSTM1/p62 (Polyclonal)	Rabbit	1:1000 in 5% BSA	CST	5114

CST, Cell Signaling Technology; DSHB, Developmental Studies Hybridoma Bank

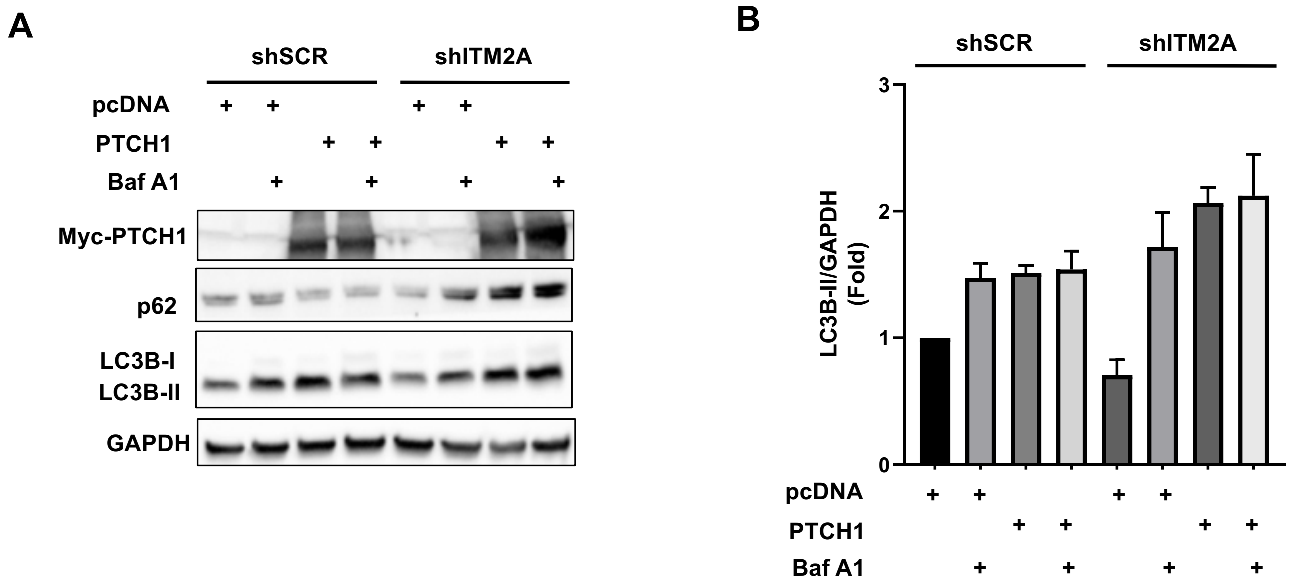

Supplementary Figure S1. Effect of ITM2A silencing on the autophagic function of PTCH1 in HeLa cells. A. Expression levels LC3B-II in shScrambled (shSCR) and shITM2A cells transfected with pcDNA or myc-PTCH1, and cultured 24 h in complete growth medium with or without the addition of 100 nM Bafilomycin (BafA1) during the last 4 h. Representative experiment of n = 3. B. Densitometric quantification of LC3BII normalized to GAPDH. Results are mean +/- SEM (n=3).

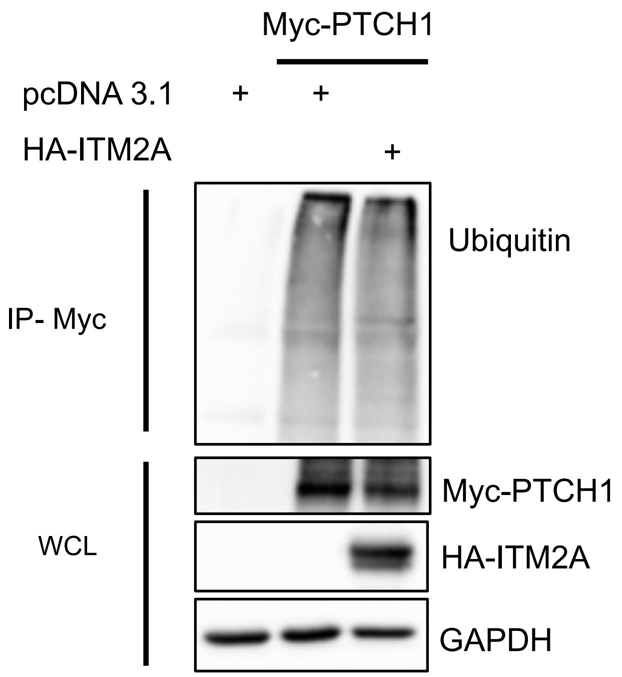

Supplementary Figure S2. Effect of ITM2A on ubiquitylation of PTCH1. PTCH1 was expressed along empty vector or ITM2A-encoding plasmid in HEK293 cells. After 20 h, MG132 was added to the medium to prevent proteasomal degradation of ubiquitylated proteins. Cells were lysed 24 h post-transfection, followed by immunoprecipitation of PTCH1-myc and blotting for ubiquitination.
